# Frailty and Parkinson’s disease: the role of diabetes mellitus

**DOI:** 10.3389/fmed.2024.1377975

**Published:** 2024-05-30

**Authors:** Klara Komici, Antonella Pansini, Leonardo Bencivenga, Giuseppe Rengo, Gennaro Pagano, Germano Guerra

**Affiliations:** ^1^Department of Medicine and Health Sciences, University of Molise, Campobasso, Italy; ^2^ASL Avellino, Avellino, Italy; ^3^Department of Translational Medical Sciences, University of Naples “Federico II”, Naples, Italy; ^4^Istituti Clinici Scientifici Maugeri IRCCS-Scientific Institute of Telese Terme, Telese Terme, BN, Italy; ^5^Roche Pharma Research and Early Development (pRED), Neuroscience and Rare Diseases Discovery and Translational Area, Roche Innovation Center, Basel, Switzerland; ^6^University of Exeter Medical School, London, United Kingdom

**Keywords:** aging, frailty, diabetes mellitus, Parkinson’s disease, insulin resistance, hyperglycemia

## Abstract

Parkinson’s disease (PD) is a chronic neurodegenerative disease associated with a progressive loss of dopaminergic neurons, clinically characterized by motor and non-motor signs. Frailty is a clinical condition of increased vulnerability and negative health outcomes due to the loss of multiple physiological reserves. Chronic hyperglycemia and insulin resistance, which characterize diabetes mellitus (DM), have been reported to alter dopaminergic activity, increase the risk of PD, and influence the development of frailty. Even though diabetes may facilitate the development of frailty in patients with PD, this relationship is not established and a revision of the current knowledge is necessary. Furthermore, the synergy between DM, PD, and frailty may drive clinical complexity, worse outcomes, and under-representation of these populations in the research. In this review, we aimed to discuss the role of diabetes in the development of frailty among patients with PD. We summarized the clinical characteristics and outcomes of patients with concomitant DM, PD, and frailty. Finally, interventions to prevent frailty in this population are discussed.

## 1 Introduction

Frailty and Parkinson’s disease (PD) are conditions frequently associated with advancing age. Frailty is a disorder of several physiological systems that implies concerns related to vulnerability and negative outcomes. The overall prevalence of frailty and prefrailty in community-dwelling adults is, respectively, 17% (95% Confidence Interval [CI] 16–18%), and 45% (95% CI 44–46%) (1). Regardless of the definition of frailty, the prevalence of frailty in community-dwelling adults across increasing age groups is progressively high. In individuals aged 60–69 years, the prevalence is 16%; in 70–79 years, 20%; in 80–89 years, 31%; and in above 90 years, 51% (1). Considering the geographic region, physical frailty prevalence appears higher in Africa and lower in Europe: 22% vs. 8%. For women, physical frailty and prefrailty prevalence proportions are 15 and 49% compared with 11 and 45% for men (1). Fatigue, weight loss, gait impairment, fluctuating disability, and confusion are common clinical presentations of frailty.

The prevalence of PD rises steadily with age: 41 per 100.000 in 40–49 years; 107 per 100.000 in 50–59 years; 428 per 100.000 in 60–69 years; 1,087 per 100.000 in 70–79 years; and 1903 per 100.000 in older than 80 years (2). Differences in prevalence by geographic location have been reported: 1601 per 100.000 in individuals of 70 to 79 years of age from Europe, North America, and Australia, compared with 646 per 100.000 in individuals from Asia (2). Differences were found for women and men of 50 to 59 years of age: 41 per 100.000 in women and 134 for 100.000 in men (2). PD is characterized by motor symptoms: bradykinesia, rigor, tremor, and postural instability. Cognitive decline, depression, sleep disorders, orthostatic hypotension, and gastrointestinal disorders are cardinal non-motor symptoms and conditions associated with PD. Hoen and Yahr (HY) scale is a clinical rating scale used to categorizing patients affected by PD. HY scale includes five stages and describes important aspects of motor involvement, compromised balance, physical independence, and disability (3). The Unified Parkinson Disease Rating Scale (UPDRS) and its modified version: Movement Disorder Society-Unified Parkinson Disease Rating Scale (MDS-UPDRS) is a comprehensive tool utilized for the quantification of PD severity and progression (4). The MDS-UPDRS covers non-motor and motor aspects of daily living, motor examination, and motor complications.

Progressive loss of dopaminergic neurons and impairment of serotoninergic signaling are considered cardinal pathways in the pathophysiology of PD (5, 6). Of note, chronic hyperglycemia and insulin resistance have been reported to alter dopaminergic activity and influence the onset of PD (7, 8). Indeed, experimental and clinical evidences have underlined common pathophysiology mechanisms between diabetes mellitus (DM) and PD (9). DM is common among the elderly, accounting for a prevalence rate of up to 30% (10). Age-dependent modification of body composition and age-related insulin resistance may influence the high incidence rate of DM among the elderly (10, 11). In addition, longitudinal studies underline that diabetes is an important factor in the progression of frailty (12, 13). Reasonably, the decline of motor functions and the onset of neuropsychiatric conditions, which characterize PD, influence the development of vulnerability and progression to frailty. It should be mentioned that frailty assessment models include diabetes as part of the deficits necessary for frailty detection. The presence of DM in studies that focus on the relationship between PD and frailty is not rare (14, 15). Even though diabetes may facilitate the development of frailty in patients with PD, this relationship is not established and a revision of the current knowledge is necessary. Furthermore, the synergy between DM, PD, and frailty may drive clinical complexity, worse outcomes, and under-representation of these populations in the research. In this review, we aimed to discuss the role of diabetes in the development of frailty among patients with PD. We summarized the clinical characteristics and outcomes of patients with concomitant DM, PD, and frailty. Finally, interventions to prevent frailty in this population are discussed.

Considering the aim of this review, an article search was performed on MEDLINE/PubMed using a combination of the following free text terms and major medical subject headings: “Frailty,” “Parkinson’s Disease,” and “Diabetes Mellitus.” We searched articles published until November 2023. Additional articles were also identified by the reference list of studies included in this review. We reviewed studies that evaluated physical and or multidimensional frailty in patients with PD and diabetes. Both experimental and clinical studies on the relationship between PD and diabetes, diabetes, and frailty were also considered for this review. Our search was limited to articles published in the English language.

## 2 The concept of frailty

Frailty has been defined as a geriatric syndrome, characterized by multidimensional loss of physiological reserves, vulnerability toward stressor events, and negative health outcomes (16). Physical, cognitive, psychological, and social functioning are considered the main domains of frailty (17). Lower physical activity is associated with frailty and disability (18, 19), whereas higher physical activity levels resulted in 41% decreased odds of frailty (20). The incidence of frailty in individuals with malnutrition is 10.35 times higher (95% CI: 3.78–28.36) than the incidence of robustness (21). A significant association between an increased number of medications and frailty has been reported (22). Comorbidities such as dementia, heart failure, and cancers are characterized by a high prevalence of frailty (23–25). Disability, reduced quality of life, falls, fractures, delirium, hospitalizations, need for long-term care, and death are negative outcomes of frailty. It has been suggested that chronic inflammation, activation of the immune system, age-related modification of the endocrine system, and nervous and cardiorespiratory systems are important factors for the development of frailty (26, 27).

Several frailty assessment tools have been described in the present literature (28). However, quantification of frailty is based on the frailty phenotype (FP) model, frailty index (FI) derived by accumulative deficits and comprehensive geriatric assessments (CGA). Fried’s FP is a physical frailty model and one of the most common frailty models applied as a prognostic tool. Unintentional weight loss of 4.5 kg or more during the last year, low handgrip strength, self-reported exhaustion, slow walking speed, and low physical activity are criteria of Fried’s FP, and the presence of three or more of them identifies frailty (29). The presence of one or two of these criteria classifies individuals in prefrail. Although a widely accepted definition of prefrailty is lacking, it has been suggested that prefrailty is a multifactorial, multidimensional, and dynamic syndrome (30). Prefrailty should be considered as a transitional and reversible state before the onset of frailty. The clinical manifestations of prefrailty are weakness, fatigue, or no symptoms.

FI based on accumulative deficits incorporates symptoms, signs, disabilities, and comorbidities (31). This model is computed by the number of health deficits divided by the total number of the variables screened. A higher number of health deficits identifies greater frailty. FI derived from comprehensive geriatric assessment includes functional, nutritional, cognitive, and psychological assessments and is highly associated with FI based on accumulative deficits (32). The Clinical Frailty Scale (31), the Edmonton Frailty Scale (33), the Study of Osteoporotic Fractures Frailty Index (34), the Tilburg Frailty Indicator (35), and the Multidimensional Prognostic Index (36) are other frailty measurement tools, which originate from the main frailty models and have been validated in different populations and clinical settings. Despite different frailty measurements having different accuracy (37, 38) the ability of frailty models in the prediction of adverse clinical outcomes has been well established (36).

Even though the impact of frailty on health outcomes is strong, frailty is not routinely measured in clinical practice. Clinical assessment of frailty status tends to be evaluated by the perception of clinicians of patient frailty, the experience of clinicians, and self-perception of patients. It has been reported that objective measurement of frailty results in different to perception of health providers and or self-perception (39, 40). Anyhow, a potential relationship between the perception of frailty and survival has been described (41).

## 3 Frailty and Parkinson’s disease

Approximately 38% of patients with PD are identified as frail by the FP model (42). The occurrence of frailty among patients with PD compared to patients without PD resulted higher in different studies: 69.4% vs. 24.2% (43) and 35.6% vs. 5.2% (44). A longitudinal study including patients with newly diagnosed PD concluded that the presence of PD increased frailty risk: odds ratio (OR) =6.68; 95% CI (3.15–15.62) (15). It should be mentioned that patients with PD compared to controls are characterized by a higher number of comorbidities and polytherapy (45). Furthermore, sarcopenia is common, and it is associated with disease severity in PD (44, 46). PD may trigger the development of frailty, but a bidirectional relationship is also possible. Frail patients, identified with either Fried’s FP or FI, had approximately 4- to 12-fold higher odds of having a diagnosis of PD diagnosis and 2.8 to 8.3 higher odds of prodromal PD (47). Furthermore, a recent prospective cohort study concluded that prefrailty and frailty were associated with incident PD, independent of genetic background, comorbidities, sociodemographic factors, and lifestyle (48).

Frailty has been associated with longer PD duration: 16.5 ± 8.5 years in frails vs. 9.6 ± 6.3 years in non-frail patients (*p* < 0.001) (44). Data from several studies report that frail patients compared to non-frail patients present a significantly higher Hoehn and Yahr Scale, indicating a more advanced stage. For instance, the Hoehn and Yahr Scale in frail vs. non-frail patients resulted: 3.3 ± 0.9 vs. 2.0 ± 0.8, *p* < 0.001 (44); 2.17 ± 1.12 vs. 1.54 ± 1.02, *p* < 0.009 (49); 2.5 ± 0.9 vs. 1.5 ± 0.6, *p* < 0.001 (50). Frailty has been associated with significantly higher scores in MDS-UPDRS part I-IV (44, 50). UPDRS parts II and III were significantly different in patients with idiopathic PD (50). Postural instability and gait disorder were more common among frail patients with PD, while tremor dominant subtype less frequent (44, 50). In contrast, a recent study observed that frail patients were characterized by a higher risk of rest tremor, facial bradykinesia, overall bradykinesia, and rigidity (47). Higher doses of levodopa therapy have been described among frail patients (44, 49, 50), and frailty is independently associated with higher levodopa doses (50).

Short-term memory, attention, visuospatial function, and executive function were significantly worse in frail patients with PD (49). Cognitive performance evaluated by Montreal Cognitive Assessment (MoCA) resulted in significant differences among frail patients with PD compared to non-frail patients: 22.6 ± 4.2 vs. 27.5 ± 2.5 (*p* < 0.001) (50). In line with these results, other studies also describe an increased risk for cognitive decline and dementia (15, 44, 51). The relationship between cognitive decline and frailty remained significant even after adjustment for potential confounders such as age, gender, PD duration, and therapy (50).

The Geriatric Depression Scale was significantly associated with frailty in patients with PD, regardless of the gravity of movement disorders (52). In line with these results, another study reported an independent association between depression and frailty in a group of patients with PD (53). In addition, disability was a significant characteristic of patients with concomitant PD and frailty. Reduced quality of life has been reported as a characteristic of frail patients with PD (44, 54, 55). However, a pilot observation study did not find a significant effect of frailty on the quality of life among PD patients, indicating that more research is necessary in this field (56). Furthermore, both self-perception of physical and mental health were related to postural control and impacted the quality of life (57). Self-perceived weakness in patients with PD without demonstrable weakness in neurological examinations was associated with fatigue, which is one of the characteristics of prefrailty (58). Self-perceived quality of mobility correlated with cerebellum hyper-metabolism and frontal hypo-metabolism as demonstrated by PET imaging, suggesting that perception of impaired quality of mobility may have a neurophysiological basis related to both motor and non-motor manifestations in PD (59).

Regarding the impact of frailty on survival, different studies have described that frail patients with PD present higher odds of in-hospital mortality and reduced overall 1-year survival (60–62). Furthermore, frail patients presented a higher risk of other adverse events such as falls, delirium, and hospitalizations (62).

## 4 Diabetes mellitus and Parkinson’s disease

The relationship between diabetes and PD has been explored by a considerable number of experimental and clinical studies. An experimental model of early type 2 DM induced by high-fat diet revealed impairment of nigrostriatal dopamine function and increased iron deposition in substantia nigra (63). Accumulation of α-synuclein and neuroinflammation were aggravated in the midbrain of type 2 DM, suggesting that metabolic inflammation exacerbates degeneration of neuronal dopamine (64). Furthermore, insulin resistance mediated the activation of reactive oxygen species (ROS), mitochondrial dysfunction, and increased α-synuclein in dopaminergic neurons (65). It has demonstrated that chronic upregulation of IL-1β and IL-18 leads to increased insulin levels, which may be important for DM development (66). In addition, alpha-synuclein deposition activates NLRP3 inflammasome via cathepsin B signaling, which, in turn, may enhance PD development (67). Recently, it has been described that the adrenergic system in specific β2-adrenergic receptors (β2AR) modulates the transcription of α-synuclein and use of β2AR agonists, such as salbutamol was associated with reduced risk of PD development (68). Of interest, experimental models have revealed that β2AR signaling regulates pancreatic β-cell insulin secretion, and silencing of the β2AR or pharmacological treatment with β_2_AR antagonist resulted in glucose response impairment. Therefore, it can be hypothesized that the implication of β2AR signaling in DM may modulate the expression of α-synuclein and trigger the development of PD.

Several clinical studies have highlighted an increased risk of PD in patients with DM (69–72). In a previous meta-analysis study, we reported that the prevalence of DM among PD patients is approximately 10% and diabetic patients suffer from a higher risk of developing PD: OR = 1.34; 95% (CI 1.26–1.43 *p* < 0.0001) (73). Furthermore, pre-diabetes increased the odds of subsequent PD and this association was more accentuated among young individuals and the female population (74). Increased glycated hemoglobin (HbA1c) levels were associated with neuroaxonal damage and cognitive impairment among patients with PD (75). However, the association of diabetes and PD is not supported by all studies. A large prospective study did not provide evidence for any relationship between baseline diabetes and risk of PD (76) and a meta-analysis study of case–control studies suggested that diabetic patients may have a decreased incidence of PD (77). It should be mentioned that the heterogeneity in cross-sectional, case–control, and cohort studies focusing on the relationship of PD and DM is high. These discrepancies may be explained also by the interaction of demographic characteristics such as age and gender. Indeed, we identified age as an important moderator of the prevalence of diabetes among PD (73). Another factor as increased medical surveillance in diabetic patients, the effect of diabetes on cardiovascular mortality, and anti-hyperglycemic agents may influence the PD-DM relationship (78). It has been reported that in patients with concomitant PD and DM, worse postural symptoms balance impairment, and faster motor progression are present (79, 80). Furthermore, diabetic patients with PD have a faster cognitive decline and impairment of attention, working memory, and frontal executive function (81, 82).

Insulin pre-treatment showed a protective effect against cell toxicity induced by 1-methyl-4-phenyl pyridinium used in PD experimental models. Insulin also ameliorated insulin signaling pathways in dopaminergic neurons (83). Intranasal treatment with insulin provided protective effects on dopaminergic neurons in a rat model of PD, in parallel with improvement in motor activity and behavior (84).

In clinical studies, a single dose of intranasal insulin increased the resting-state functional connectivity between the default multiple network and hippocampal regions in older adults with type 2 DM (85). Another study demonstrated that in diabetic patients, intranasal insulin enhanced vasodilatation in the insular cortex, which regulates task performance related to attention (86). In persons with Alzheimer’s disease, intranasal insulin administration did not show effects on cognitive and functional performance over a period of 12 months (87). However, data from a pilot longitudinal study report that in PD, intranasal insulin administration may improve functional motor skills and may preserve cognitive performance (88).

The incident rate of PD in a cohort of metformin exposure was 5.9 cases per 1,000 patients per year, compared to 2.43 cases per 1,000 patients per year in the group without metformin exposure. More than 4 years of metformin exposure was associated with a lower risk of PD: adjusted HR = 0.04; (95% CI 0.00 to 0.37) (89). In contrast, a recent analysis concluded that metformin use was associated with a significantly increased risk of PD incidence OR = 1.66, (95% CI 1.14–2.42), compared with non-metformin users or glitazone therapy (90). In a Taiwanese population cohort, sulfonylureas increased the risk of PD by 57%, and combination with metformin use avoided this effect (91). Overall, a meta-analysis study did not find any change in the risk of PD related to sulfonylurea administration: HR: 1.13 95% CI: 0.96–1.32 (92). An inverse association between the use of thiazolidinediones and an incidence of PD, with an HR of 0.74 (95% CI, 0.59–0.92) has been described (93) and another observation found a slight reduction of PD risk (94). However, a nationwide population-based study did not find a beneficial role (72).

Preclinical studies have reported that glucagon-like peptide-1 receptor (GLP1R) agonists improved dopamine levels and reduced neuronal damage through the modification of oxidative stress and inhibition of inflammatory cytokines (95). A preliminary data analysis reported that diabetic patients treated with GLP1R agonists had a significant improvement at 12 months on the MDS-UPDRS of 2.7 points, compared with a mean decline of 2.2 points in control patients (*p* = 0.037) (96). A significantly reduced rate on the onset of PD and use of GLP1R agonists has been also described as incidence rate ratio (IRR) =0.38 (95% CI 0.17–0.60; *p* < 0.01) (97). However, results from another study did not show a significant association (94), and the response to therapy was worse in elderly patients with DM longer than 10 years (98). For the first time, a nationwide case–control study reported that the use of dipeptidyl peptidase-4 (DPP4) inhibitors was associated with a decreased risk of PD: OR = 0.23 (95% CI 0.07–0.74) (99). Strong evidence of an inverse association between the use of DPP4 inhibitors and the incidence of PD was described by another study IRR = 0.64; (95% CI 0.43–0.88; *p* < 0.01). Finally, a meta-analysis of studies reporting data on (DPP4-i) reveals that DPP4 inhibitors use was associated with reduced risk of PD: HR:0.69 95%CI:0.56–0.86 (92).

Modification of insulin secretion in pancreatic cells after levodopa therapy has been described in a rodent model (100). Furthermore, bromocriptine, a dopamine agonist, improved glycemic control and reduced insulin requirement in type 2 DM subjects on high-dose insulin therapy (101). Meta-analysis of randomized controlled trials found that dopamine agonists improve glycemic control in diabetic patients without serious adverse events (102). A large primary care-based national observational study observed that the incidence of diabetes in patients with PD occurred less frequently: OR = 0.53 (95% CI 0.33–0.87). The odds of developing diabetes in patients with PD and levodopa therapy were higher compared to patients without PD and levodopa therapy: OR = 0.22 (95% CI 0.10–0.48) (103).

## 5 Diabetes mellitus and frailty

Frailty and DM share common pathological mechanisms. Age-dependent reduction in skeletal muscle mass, sarcopenia, and increased visceral adiposity is associated with insulin resistance (104, 105). Oxidative stress, mitochondrial dysfunction, and chronic inflammation are other mechanisms linked to both frailty and diabetes (10).

Analysis adjusted for potential age, gender, and other confounding risk factors resulted in a consistent association between DM and frailty prevalence (106). DM has also been associated with a lower likelihood in the improvement of frailty status (107). Hyperglycemia has been associated with the development of frailty. Furthermore, a U-shaped relationship between glycemia and frailty has been described where glycemia levels <8.8 mmol/L and > 10 mmol/L were associated with an increased risk of frailty (108). Hypoglycemia and glycemic decompensation were associated with multidimensional impairment in the elderly with DM (109). However, a recent study found that out-of-range glucose concentration, defined also as dysglycemia, is significantly associated with incremental frailty, and hyperglycemia was predictive of mortality explainable by frailty (110). It has been reported that patients with higher Hb1Ac at baseline developed worse frailty status during 10 years of follow-up (111). However, another study did not find a U-shaped relationship between frailty and HbA1c level, suggesting that good glycemic control might be more important for frailty than poor glycemic control in patients with DM (112).

Patients with DM and frailty, regardless of methods used to quantify or measure frailty, are characterized by an increased risk of overall mortality compared to non-frail patients with DM (106).

Diabetic patients are characterized by a high risk of fractures despite normal or increased bone mineral density (113). Of interest, a prospective cohort study revealed a significant relationship between the risk of incident fragility fractures and frailty: HR of 1.02 (95% CI 1.01–1.03) (114).

Cardiovascular, cancer-related, and all-cause mortality were higher among middle-aged adults with DM. Furthermore, falls, major cardiovascular events, and hypoglycemia were also significantly related to the presence of frailty (115). Of note, in frail diabetic and hypertensive patients, a significant interaction between physical and cognitive domains has been described (116). Evaluations of cognitive status and physical performance with 5-min walking speed test would be useful for the elderly with DM or other cardiovascular risk factors. Furthermore, insulin resistance was a significant and independent predictor of cognitive performance in prediabetic frail patients (117). Considering the strict correlation between physical and cognitive performance the concept of cognitive frailty has been proposed, cognitive frailty has been defined as the coexistence of physical frailty and cognitive impairment in the absence of other neurological diseases and/or Alzheimer’s disease (118).

A recent study reported that cognitive frailty is common among diabetic patients. Age, duration of diabetes, intellectual activity, albumin levels, calf circumference, and depressive state were identified as independent risk factors (119). Furthermore, closer attention to the elderly who have poor self-care ability and low income has been suggested as early indicators of cognitive frailty in diabetic patients (120).

Management of elderly people with DM is complex as a consequence of multimorbidity, polytherapy, and complications related to adverse drug events and hypoglycemia. Recent consensus statements on the management of elderly with type 2 DM indicate frailty assessment as a component of the clinical management and modification of glycemia targets. Therapeutic choices should be tailored to vulnerability and frailty status (121). An HbA1c target of 64–69 mmol/mol is indicated in patients with severe frailty and reduced life expectancy (122).

It has been suggested that metformin may be a potential pharmacological intervention that modifies the trajectories of frailty (123). Even though only intensive lifestyle interventions reduced frailty prevalence among frail diabetic patients and metformin use was not associated with significant reduction (124), administration of extended-release metformin in frail women with concomitant diabetes and hypertension ameliorated cognitive performance (125). In line with this finding, during a 4-year follow-up study, metformin use was associated with a reduction of frailty risk (126).

Frailty was associated with a significantly lower probability of initiating therapy with a GLP-1 receptor agonist and an SGLT2 inhibitor than non-frail diabetic people (127). Empagliflozin reduces frailty in diabetic and hypertensive patients, most likely by reducing mitochondrial Ca2+ overload and reactive oxygen species (128). Recently, another study concluded that GLP1R agonists and SGLT2 inhibitors safely improved cardiovascular outcomes and all-cause mortality, with higher benefits among frail patients (129).

## 6 Frailty and Parkinson’s disease: the potential role of diabetes mellitus

A case–control study that included patients with a diagnosis of DM prior to PD showed that diabetic patients with PD require higher doses of levodopa treatment and experience more severe PD symptoms (129). Other studies have described that higher doses of levodopa were associated with frailty, regardless of the model used for frailty assessment (44, 50, 53). In contrast, another study reported that the median annual levodopa equivalent dose increased initially in the non-frail (400 mg/day) and prefrail groups (439 mg/day). Levodopa equivalent dose declined progressively in the mildly frail 400 mg/day, moderately frail (334 mg/day), and severely frail (304 mg/day) groups (61). The relationship between frailty and drug therapy is bidirectional, and medication review is necessary for the frail patients, and therefore, underestimation of the levodopa–frailty relationship may be influenced by dose modifications. Diabetic patients with PD, compared to non-diabetic patients with PD, differed significantly regarding cognitive performance, behavioral and mood disorders, and activities of daily living. In addition, motor examination showed worse outcomes in patients with DM compared to patients not affected by DM (129). Prospective studies focused on the relationship between incident frailty and depressive symptomatology provide evidence for increased bidirectional risk (130). Depression occurs in approximately 25–50% of patients affected by PD. Chronic inflammation and increased cortisol levels may lead to insulin resistance and impairment of the hypothalamic–pituitary–adrenal axis (131). Furthermore, depression, cognitive decline, and motor symptoms resulted in significant predictors of impaired activities of daily living and the development of dependency among patients with PD (132). Another case–control study found that diabetic patients with PD are characterized by increased postural instability and motor feature severity. Postural instability persisted even after controlling for hypertension and BMI. These clinical features were not explained by differences in striatal dopaminergic denervation, leukoaraiosis, or large-fiber polyneuropathy (133). Postural instability determines a greater risk of developing prefrailty and frailty among the elderly (134). Gait impairment, beyond gait speed, could help identifying different categories of frailty. It has been suggested that gait variability might reflect a multidimensional reduction and may be useful in identifying frailty (135).

Progression in the severity of UPDRS part III motor signs such as gait impairment and bradykinesia together with a significant overall cognitive decline have been associated with diabetes in PD patients (136, 137). While this study did not observe differences in specific memory domains across PD patients with and without DM, another study (136) underlined that frontal executive functions and attention were impaired in this population (81). Impairment in executive functions resulted in an independent risk factor for the development of physical frailty in patients with PD. Executive functions coordinated by the prefrontal cortex and subcortical areas are necessary to perform complex tasks or activities. Loss of the dopaminergic neurons and a disconnected hippocampus, amygdala, and prefrontal cortex may influence cognitive function, gait speed, and muscle mass loss.

**Figure 1:**
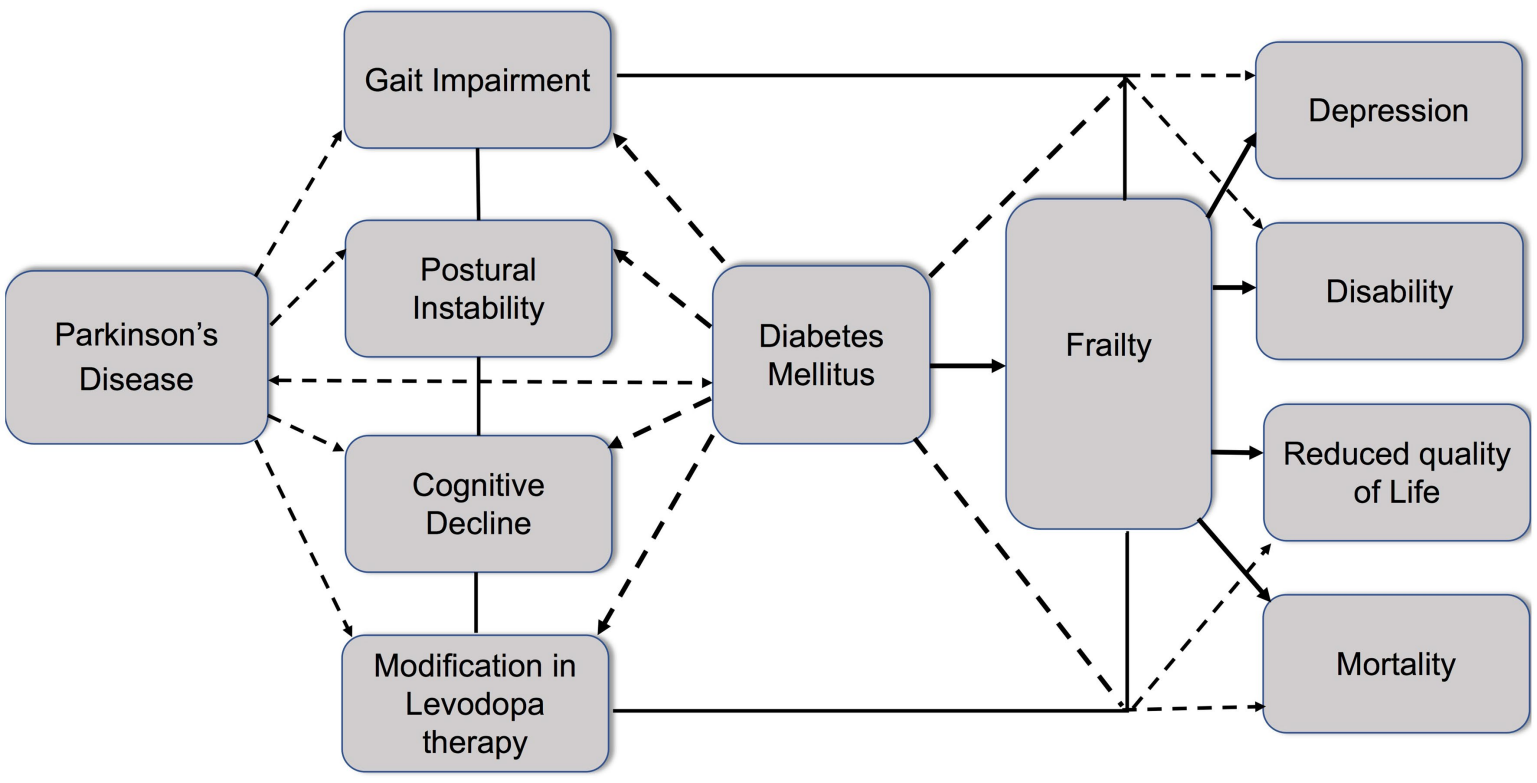
Schematic diagram highlighting the relationship between diabetes, Parkinson’s disease, and frailty. Parkinson’s disease (PD) is characterized by gait impairment, postural instability, and cognitive decline. Modification of levodopa therapy may be necessary during PD progression. Diabetes mellitus (DM) and PD share common pathomechanisms. Diabetes increases the risk of PD onset and aggravates symptoms such as gait impairment, postural instability, cognitive decline, and the need for modification in levodopa therapy. Moreover, diabetes contributes to the development of frailty in PD patients, leading to the enhancement of negative outcomes, including depression, disability, reduced quality of life, and increased mortality.

Sarcopenia and PD share common characteristics (138). Patients with PD are characterized by poor physical performance and lower physical activity compared to healthy population. Poor nutritional status, modification in body composition, and hormonal axis alternations may influence the development of sarcopenia in PD. Furthermore, degeneration of the motor neuron units, reduction in the number of motoneurons, and modification of the gray matter have been suggested as possible mechanisms in patients with PD who have sarcopenia (139, 140). Finally, diabetic neuropathy may affect muscle strength and insulin resistance and chronic hyperglycemia may lead to a reduction in muscle mass and hand grip strength and physical performance (141). The relationship between DM PD and frailty is presented in Figure 1.

### 6.1 Interventions to prevent frailty among diabetic patients with Parkinson’s disease

Implementation of routine assessments for the identification and stratification of frailty status is of great importance considering the overall negative impact on health outcomes. Early detection of prefrailty and or frailty in diabetic patients affected by PD would be necessary to develop specific and tailored interventions in order to reduce the disability and dependence burden. On the other side, screening for DM should be regular and accurately performed also in frail patients with PD.

Physical activity is considered a plausible protective factor for both DM and PD. Aerobic activity enhanced cognitive performance and stabilized the progression of PD in the corticostriatal sensorimotor network (142). Balance and strengthening training were not effective in reducing repeat falls across patients with PD, but balance and self-efficacy significantly improved. Furthermore, this intervention may be more beneficial among patients with moderate PD (143). However, another study found that after 10 weeks of exercise, the self-perceived fall risk improved only in severe PD (144).

Modest increments in moderate to vigorous physical activity had a clinically meaningful impact regarding cardiovascular risk factors and scores in diabetic patients (145). Future studies should evaluate the role of tailored training programs in diabetic patients with PD.

Several clinical investigations have reported that DPP4-i and GLP1R agonists were associated with reduced risk of PD (92, 96, 97) and also a beneficial role in frailty status have been associated with GLP1R agonists and sodium-glucose cotransporter 2 (SLGT2) inhibitors (128, 146). Indeed, experimental models reveal that treatment with dulaglutide, a GLP1R agonist, is protective against skeletal muscle injury by inhibiting inflammation and regulating the differentiation of myoblasts (147). A retrospective longitudinal analysis revealed that basal insulin co-therapy and GLP-1 receptor agonists may be effective in maintaining appendicular skeletal muscle mass (148). Future studies should investigate the role of antidiabetic agents not only in the prediction but also amelioration of frailty status among diabetic patients with PD.

## 7 Conclusion

The current evidence suggests that diabetic patients with PD have an increased risk for the development of frailty. Frail patients with concomitant PD and DM are characterized mostly by gait impairment, postural instability, sarcopenia, and cognitive decline. Dependency, depression, low quality of life, higher doses of levodopa therapy, and an overall negative health outcome may further characterize PD patients with DM. Early detection of vulnerability and frailty status and glycemic control in this population would be necessary for a better management of patients. Future research should explore the impact of interventions tailored to frailty aspects on health outcomes. Specific mechanisms of insulin resistance that contribute to frailty and the role of antidiabetic drugs on frailty among diabetic patients with PD should be investigated. Clinical trials should evaluate the role of antidiabetic drugs in the prevention and amelioration of frailty among diabetic patients with PD.

## Author contributions

KK: Conceptualization, Writing – original draft, Writing – review & editing. AP: Writing – original draft, Writing – review & editing. LB: Writing – original draft, Writing – review & editing. GR: Writing – original draft, Writing – review & editing. GP: Writing – original draft, Writing – review & editing. GG: Writing – original draft, Writing – review & editing.
